# Supplementary material for: The High-Efficiency Design Method for Capacitive MEMS Accelerometer
Source: Micromachines (Basel). 2023 Sep 30;14(10):1891. doi: 10.3390/mi14101891 (PMC10609016; doi:10.3390/mi14101891)
Supplement: Supplementary file 1 [file micromachines-14-01891-s001.zip › micromachines-2560425-supplementary.pdf]

micromachines

Article

The High-Efficiency Design Method for Capacitive MEMS Accelerometer

Wen Liu [1], Tianlong Zhao [1,2,*], Zhiyuan He [1], Jingze Ye [1], Shaotong Gong [1], Xianglong Wang [1] and Yintang Yang [1]

[1] School of Microelectronics, Xidian University, Xi'an 710071, China; 22111213773@stu.xidian.edu.cn (W.L.); zyhe111111@outlook.com (Z.H.); 23111213537@stu.xidian.edu.cn (J.Y.); 23111213666@stu.xidian.edu.cn (S.G.); xlwang_312@stu.xidian.edu.cn (X.W.); ytyang@xidian.edu.cn (Y.Y.)
[2] State Key Laboratory of Crystal Materials, Shandong University, Jinan 250100, China
* Correspondence: zhaotl@xidian.edu.cn

Support

From the working mechanism part in the Part 2, it is easy to know that the direct impact factors of ΔC are proof mass M, overlapping area A of adjacent electrodes, the initial gap d_0 between adjacent electrodes, spring coefficient K and acceleration a. And M, A and K are determined by many other structural parameters. For example, the length and the width of spring both influence its elastic capacitance, so the variations of these parameters lead to the changing spring coefficient K. To obtain the optimum structural parameters, it is necessary to research on the influences of every parameter and determine their values basing on that final calculation results whether could obtain the preliminary goals. COMSOL provides parameter sweep analysis, by which the influence of different parameters could be directly reflected through the calculation results. However, it is quite difficult to realize the sweep calculation covering all relevant parameters at the same time, as there are too many structural parameters available. Therefore, control variate method is adopted at sweep calculation.

Before sweep calculation and analysis, to ascertain the scales and steps of all relative parameters is quite necessary. To make sure structural rationality, parameters' ranges depend on the primary structural size of the model in this article. And to show the influence of various parameters clearly, their sweeping scales are determined as large as possible. A good example is that the initial gap between adjacent electrodes is 1 μm. To avoid the direct touch between the electrodes and gain the changing tendency as comprehensive as possible, the scale of gap d_0 is set as [0.6,1.4] μm and step is 0.1 μm. The physical meaning of each parameter is introduced in Part 2 of the text. And the sweep scales, steps and results are shown in Table S1, Figure S1 and Figure S2 below. Hereby specifically stated that the calculation result accuracy of whole mass M_a is limited by FEM software for w_{conn}, but this will not interfere with the analysis results as its values and variations are both very small.

Citation: Liu, W.; Zhao, T.; He, Z.; Ye, J.; Gong, S.; Wang, X.; Yang, Y. The High-Efficiency Design Method for Capacitive MEMS Accelerometer. *Micromachines* **2023**, *14*, 1891. https://doi.org/10.3390/mi14101891

Academic Editor: Ha Duong Ngo

Received: 31 July 2023
Revised: 10 September 2023
Accepted: 29 September 2023
Published: 30 September 2023

Copyright: © 2023 by the authors. Licensee MDPI, Basel, Switzerland. This article is an open access article distributed under the terms and conditions of the Creative Commons Attribution (CC BY) license (https://creativecommons.org/licenses/by/4.0/).

Table S1. Sweep scale and step of the other eight parameters.

Parameter	Scale(μm)	Step(μm)
d_0	[0.6,1.4]	0.1
w_{eh}	[2,6]	0.5
l_{sp}	[220,300]	10
w_{sp}	[1.2,2.8]	0.2
l_{ovrlp}	[95,103]	1
d_{sp}	[0.5,1.5]	0.1
w_f	[3.1,4.1]	0.1
w_{conn}	[2,4]	0.2

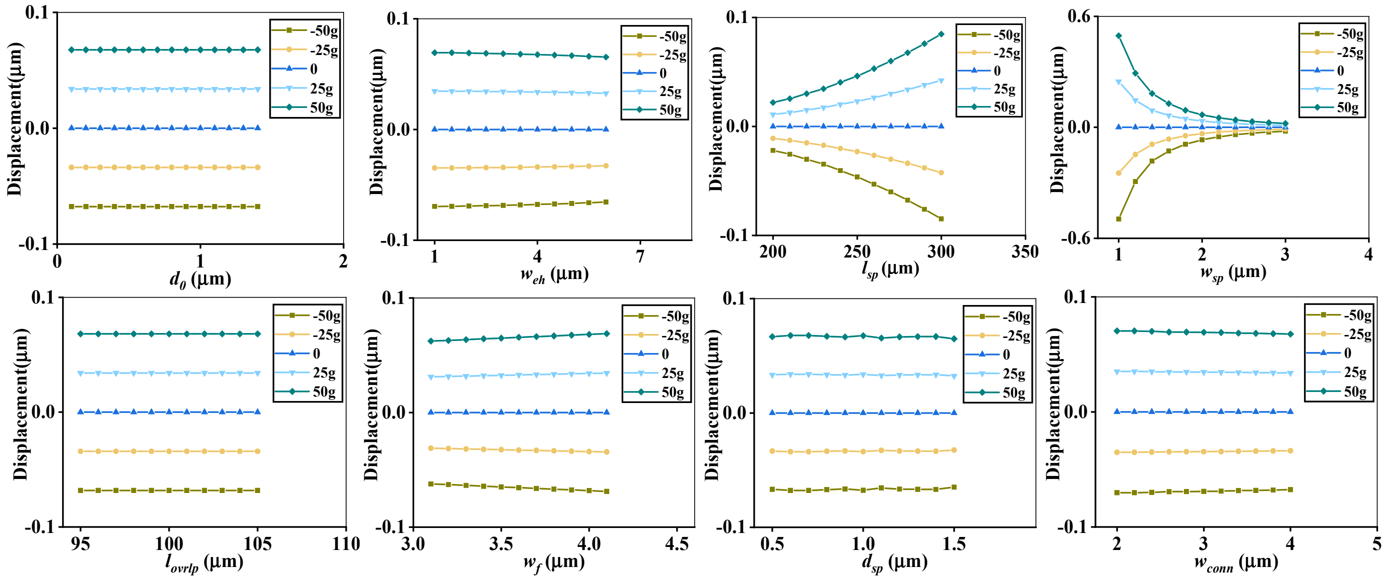

Figure S1. Displacement sweep results of the other eight parameters.

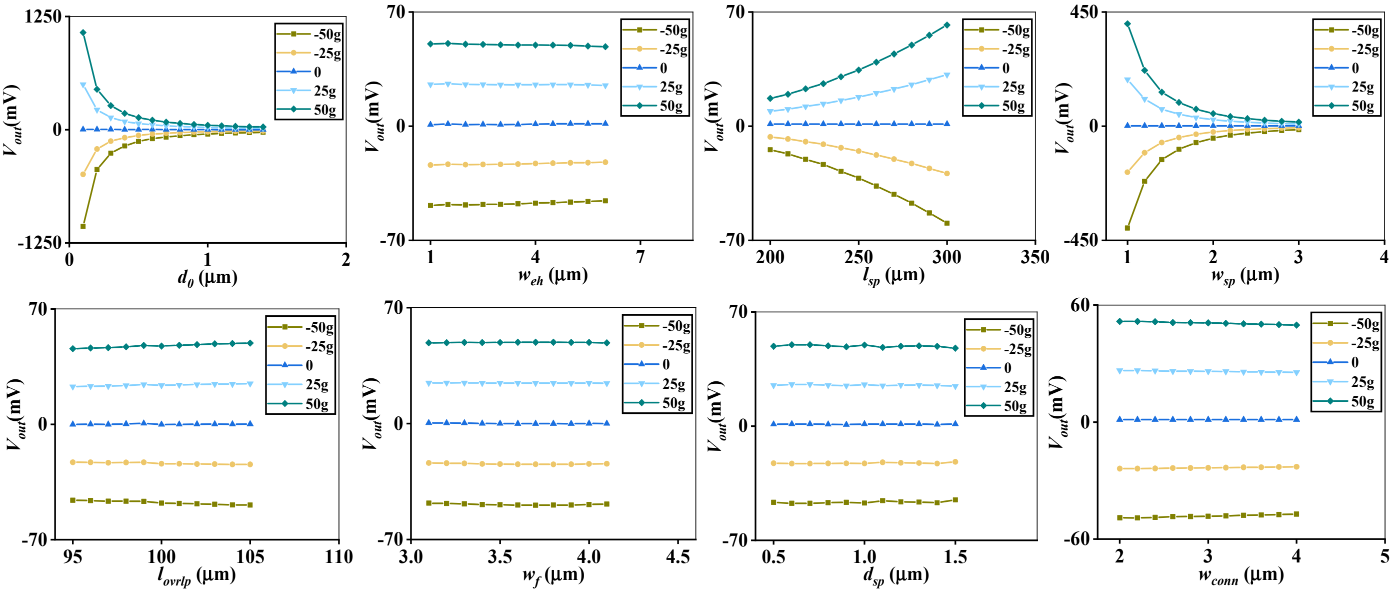

Figure S2. Output voltage V_{out} sweep results of the other eight parameters.

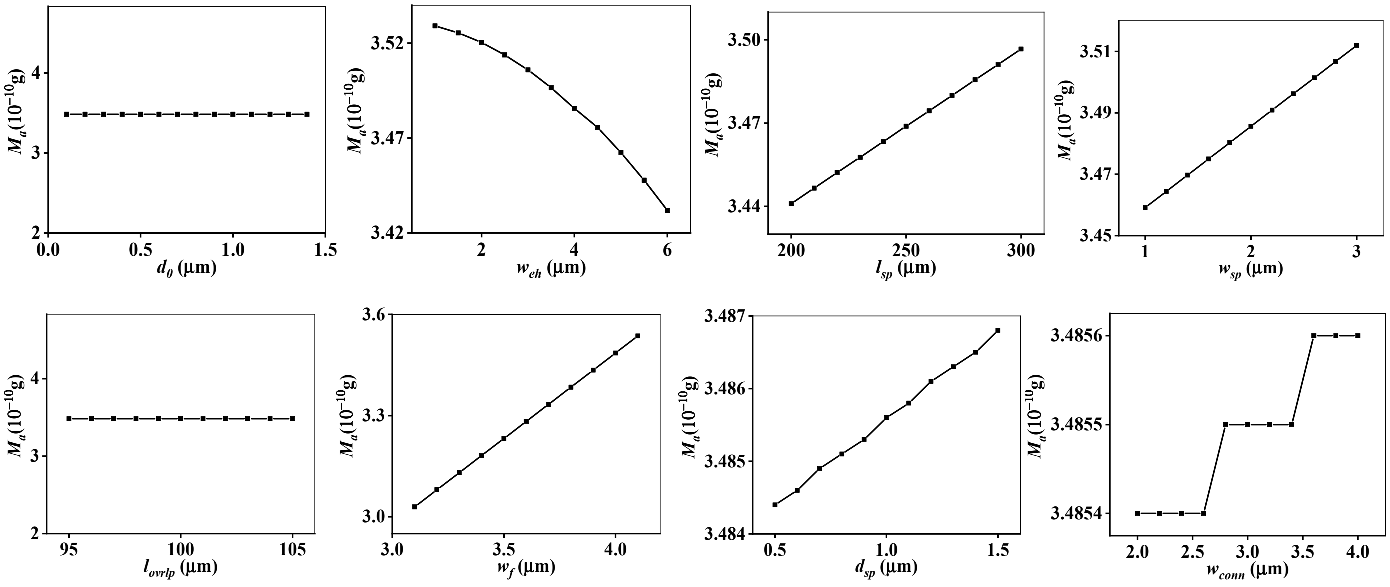

Figure S3. Overall mass M_a sweep results of the other eight parameters.
